# Negative Impact of Female Sex on Outcomes from Repetitive Mild Traumatic Brain Injury in hTau Mice Is Age Dependent: A Chronic Effects of Neurotrauma Consortium Study

**DOI:** 10.3389/fnagi.2017.00416

**Published:** 2017-12-22

**Authors:** Scott A. Ferguson, Benoit C. Mouzon, Cillian Lynch, Carlyn Lungmus, Alexander Morin, Gogce Crynen, Benjamin Carper, Gayle Bieler, Elliott J. Mufson, William Stewart, Michael Mullan, Fiona Crawford

**Affiliations:** ^1^Roskamp Institute, Sarasota, FL, United States; ^2^James A. Haley Veterans' Hospital, Tampa, FL, United States; ^3^RTI International, Research Triangle Park, NC, United States; ^4^Department of Neurobiology, Barrow Neurological Institute, Phoenix, AZ, United States; ^5^Queen Elizabeth Glasgow University Hospital, Glasgow, United Kingdom; ^6^Institute of Neuroscience and Psychology, University of Glasgow, Glasgow, United Kingdom

**Keywords:** TBI, hTau, tau, neurobehavior, Neuropathology

## Abstract

Traumatic brain injury (TBI) is a serious public health concern which strikes someone every 15 s on average in the US. Even mild TBI, which comprise as many as 75% of all TBI cases, carries long term consequences. The effects of age and sex on long term outcome from TBI is not fully understood, but due to the increased risk for neurodegenerative diseases after TBI it is important to understand how these factors influence the outcome from TBI. This study examined the neurobehavioral and neuropathological effects of age and sex on the outcome 15 days following repetitive mild traumatic brain injury (r-mTBI) in mice transgenic for human tau (hTau). These mice express the six human isoforms of tau but do not express endogenous murine tau and they develop tau pathology and memory impairment in an age-dependent manner. After 5 mild impacts, aged female mice showed motor impairments that were absent in aged male mice, as well as younger animals. Conversely, aged female sham mice outperformed all other groups of aged mice in a Barnes maze spatial memory test. Pathologically, increases in IBA-1 and GFAP staining typically seen in this model of r-mTBI showed the expected increases with both injury and age, but phosphorylated tau stained with CP13 in the hippocampus (reduced in female sham mice compared to males) and PHF1 in the cortex (reduced in female TBI mice compared to male TBI mice) showed the only histological signs of sex-dependent differences in these mice.

## Introduction

Traumatic brain injury (TBI) contributes to in excess of 2.5 million emergency department visits a year in the United States (CDC), with a 70% increase in attendances from 2001 to 2010 (Faul et al., 2010). Notably, there is a distinct sex bias in TBI presentations, with the majority of hospitalized TBI occurring in women (National Hospital Discharge Survey 2001–2010), particularly in the elderly (Stevens and Sogolow, 2005). In a meta-analysis of the influence of sex on TBI it was reported that, compared to men, women display worse outcomes from TBI across multiple measures, including mortality, days of post-traumatic amnesia, memory impairment, and insomnia (Farace and Alves, 2000), with one study of 306 individuals with a history of moderate to severe TBI documenting an increased incidence of self-reported headaches, dizziness, and depression in women with a history of TBI (Colantonio et al., 2010). Nevertheless, despite this bias, there has been limited enquiry into the influence of sex on acute or long-term consequences of TBI.

Pre-clinical research has not yet addressed potential sex-dependent differences and typically focuses on TBI in male subjects. Treatments that may be beneficial in one sex may not benefit males and females equally, or may be modulated by additional factors that need to be understood prior to attempting clinical translation. For example, environmental enrichment has been studied for its potential therapeutic benefit following TBI in rats since 1996, but until 2002 these studies were performed exclusively with male animals (Hamm et al., 1996). In 2002, a study by Wagner and colleagues demonstrated a beneficial effect of environmental enrichment in male rats which did not occur when female rats were utilized (Wagner et al., 2002). However, when the rotarod testing was performed to assay the effects of environmental enrichment, benefits were seen in both male and female rats after controlled cortical impact (Monaco et al., 2013). These results highlight the importance of understanding the differences between TBI recovery and outcome in male and female animals as these effects may impact the efficacy of experimental treatments following TBI.

The mechanisms by which sex differences influence outcome from TBI and the response to TBI treatments has not been well-studied, though studies have examined the possibility of using exogenously administered gonadal steroids as a treatment for TBI. Based on some preliminary data showing better outcome in females after TBI, a number of studies have examined potential neuroprotective effects of female sex hormone administration on the consequences of experimentally induced TBI (Behl et al., 1995; Pelligrino et al., 1998; Shi et al., 1998; Behl, 2002; Wagner et al., 2004; Xiong et al., 2007). Pre-clinical studies have shown that progesterone reduces cerebral edema (Roof et al., 1992, 1994; Wright et al., 2001; Guo et al., 2006) and estrogen maintains cerebral blood flow after experimental TBI (Roof and Hall, 2000a), but despite these pre-clinical protective effects, attempts to translate these results into human clinical trials have not been successful (Skolnick et al., 2014; Howard et al., 2016; Schumacher et al., 2016).

The disconnection between pre-clinical neuroprotective effects of female sex hormones, and their translation to clinical trials in humans may be due in part to an insufficient understanding of the role of sex in TBI and how it interacts with other biological factors such as age. For example, in clinical trials testing progesterone administration after TBI, the inclusion criteria for each trial involved patients spanning a very wide range of age at TBI of 18–60 years of age or more, and heterogeneity of the patient population of these studies has been cited as a concern for the ability of the trial design to yield relevant results (Ma et al., 2012). Both pre-clinical research in animals and clinical research in humans have shown an age-related risk for neurodegenerative disorders and a linear increase in the risk of mortality and poor outcome following TBI, but the mechanisms by which age at the time of TBI plays a role in influencing outcome from TBI have not been elucidated and age remains an under-studied factor in TBI (Hukkelhoven et al., 2003; Uryu et al., 2003; Stocchetti et al., 2012; Gardner et al., 2014). Women are known to be disproportionately represented in Alzheimer's disease (AD) patient populations, where they comprise approximately two thirds of the AD patient population in the United States (Brookmeyer et al., 1998). In contrast to this, there are very few reports in the literature of chronic traumatic encephalopathy (CTE) in women (Roberts et al., 1990; Hof et al., 1991). This may be a selection bias as most of the donated brains have been from males, but more research is needed to rule out the possibility of a sex-dependent effect on the risk for developing CTE.

In the current study, we utilize our preclinical mouse model of repetitive mild TBI (Mouzon et al., 2012, 2014; Ojo et al., 2013) to study the roles of age and sex on behavioral and pathological outcomes after repetitive mild brain injury. We have conducted these studies in mice transgenic for all six isoforms of human tau protein (hTau). Tau pathology has been documented in brain of humans after severe TBI and in sport athletes who sustained a number of concussions/mild TBI resulting in CTE, (McKee et al., 2009) and as the precise role of tau in TBI pathogenesis is not yet clear we wanted to mimic human patients as closely as possible with our model. Moreover, the interaction of sex and age with tau pathology after TBI is an area that has not been investigated before, although Tau aggregation after TBI is associated with impaired glymphatic function, which also reduces with age (Iliff et al., 2014; Kress et al., 2014). Since the role and interaction of sex and age on TBI has not been thoroughly investigated in the literature, it is important that we improve our understanding of the interaction of these factors to inform future attempts to translate potential treatments into the clinic.

## Materials and methods

### Animals

Young (aged 8–10 weeks) and aged (aged 11 to 12 months) male and female mice, expressing all six isoforms of human tau (hTau) on a C57BL/6 and null murine tau background (Jackson Laboratories, Bar Harbor ME), were housed singly under standard laboratory conditions (23°C ± 1°C, 50 ± 5% humidity, and 12-h light/dark cycle) with free access to food and water throughout the study. All procedures were carried out under Institutional Animal Care and Use Committee approval and in accordance with the National Institutes of Health Guide for the Care and Use of Laboratory Animals.

### Injury groups and schedule

Forty-eight young and 52 aged hTau mice were randomly assigned to TBI or sham conditions (unless otherwise noted, young male TBI *n* = 11, young female TBI *n* = 12, young male sham *n* = 11, young female sham *n* = 11, aged male TBI *n* = 11, aged female TBI *n* = 14, aged male sham *n* = 12, aged female sham *n* = 16). Histochemical studies were performed with *n* = 4 per group (male TBI, female TBI, male sham, female sham, for both aged and young mice). Mice assigned to repetitive mild TBI (r-mTBI) conditions received five injuries over a 9 day period, with an inter-injury interval of 48 h. Sham (r-sham) animals underwent anesthesia as per their r-mTBI counterparts (frequency and exposure time), but were not exposed to injury.

### Injury protocol

Mice were subjected to closed, mild TBI as previously described (Mouzon et al., 2012). Mice were anesthetized with 1.5 l/min of oxygen and 3% isoflurane prior to mTBI. The top of the head was then shaved for both sham and TBI animals, followed by transfer to a stereotaxic frame (Just For Mice™ Stereotaxic, Stoelting, Wood Dale, IL). Animals were placed on a heating pad to maintain body temperature at 37°C. A 5 mm blunt metal impactor tip was retracted and positioned midway relative to the sagittal suture before each impact. The injury was triggered using the myNeuroLab controller at a strike velocity of 5 m/s, strike depth of 1.0 mm, and dwell time of 200 milliseconds. The impact occurred over intact skin and produced no skull fractures, hematomas, or other gross signs of pathology. No mortality was observed with these mice during these experiments. At the end of the procedure, each animal was removed from the stereotaxic table, allowed to recover on a heating pad and, becoming ambulatory, was returned to its home cage. To control for the effects of repeated anesthesia sham animals underwent the same procedures and were exposed to anesthesia for the same length of time as the mTBI animals, but did not receive a hit.

### Neurobehavior

Experimenters were blinded to group assignments during all neurobehavioral testing. Rotarod testing was performed as previously described with modifications (Mouzon et al., 2012). One day of pre-training and baseline testing was performed on the day prior to the start of the injury or sham procedure. All mice were given 3 trials to acclimate to the Rotarod at a constant speed of 5 revolutions per minute (RPM). Following the acclimation trials, the Rotarod was set to an accelerating speed of 5–50 RPM over a period of 5 min for baseline testing and post-injury testing. One day after the administration of the last r-mTBI or r-sham procedure, Rotarod testing was resumed. Mice were then tested every other day after surgery starting on day 1 and ending on day 7 (total of 4 trials), at an accelerating speed of 5–50 RPM over a period of 5 min. Three trials were given with a 3 min rest period between trials. Latency to fall and the speed of the Rotarod at the moment of the fall was recorded for each trial and the latency to fall was averaged within each day. The post-baseline daily latency to fall averages were then reported as a proportion of baseline. Descriptive statistics, including means and standard errors, were calculated from the percent baseline fall latency times for each group and sex at each post-baseline study time for both young and aged mice. Separately for young and aged mice, the Rotarod data were fit to the following linear mixed model:

$$Fall\;Latency_{ijk}=\mu +group_{i}+sex_{j}+group_{i}*sex_{j}+\varepsilon_{ijk}$$

where μ is the overall mean percent baseline fall latency time, *group*_*i*_ is the effect of the *i*th group (*i* = mTBI or Sham), *sex*_*j*_ is the effect of the *j*th sex (*j* = female or male), *group*_*i*_ * *sex*_*j*_ is the interaction effect between *group*_*i*_ and *sex*_*j*_, and ε_*ijk*_ is the leftover difference of the *k*th subject that is unexplained by the model. In addition to these model terms, an auto-regressive [AR(1)] repeated measure for study day within an animal was included such that multiple trials on the same mouse were considered together and correlated with respect to time and not independently. This model was used to estimate the size and significance of the difference in average percent baseline fall latency between injury groups (both overall and within sex) and between sexes (both overall and within injury group).

Following Rotarod, Barnes maze testing was administered as previously described immediately after the first rotarod test (Mouzon et al., 2012). Each mouse was given 90 s to explore the maze and enter the goal box. On the 7th day the goal box was removed and the mouse was given a 60 s probe trial starting in the center of the table. Each mouse's orientation and movement was recorded by Noldus Ethovision XT software. The primary outcome in the Barnes maze data is time (in seconds) to reach the goal box. The effects of interest in this analysis were once again sex, injury group (TBI vs. Sham), and their interaction.

Since time-to-target data tends not to be normally distributed and contains ceiling effects from mice reaching the maximum trial time, the behavioral outcome was evaluated using survival analysis methods. Here, survival time, or equivalently event time, is the time (in seconds) to reach the goal box. Comparisons of event time distributions are superior to comparisons of event rates in the presence of censoring, and they have the added advantage that they are sensitive to differences in both the time to event as well as the overall percentage of events between groups.

For both young and aged mice, the event time distributions (proportion of animals in each group that have not reached the target hole at any given time) and median time-to-target were estimated within sex and injury group using Kaplan-Meier methods. The median time-to-target is the time at which half of the animals have reached the goal box. For time-to-target data, the median is preferred to the mean for two reasons: (1) censoring biases the mean and (2) time-to-target data tends not to be normally distributed.

Next, the Cox proportional hazards regression model was used to test the effects of sex, injury group, and their interaction on time to reach the goal box for young and aged mice separately. The sex by injury group interaction effect, the main effects of sex and injury group, and the effect of injury group within sex, and sex within injury group were also tested. Wald chi-square test statistics were used to evaluate the significance of model parameters. For a single degree-of-freedom hypothesis (TBI vs. sham), the Wald chi-square test statistic reduces to a standard normal deviate, or the regression coefficient estimate divided by its standard error. The estimated hazard ratio is computed by exponentiating the regression coefficient for the group effect, and it loosely represents the relative risk of reaching the goal box at any given time in the TBI vs. sham group. Values <1 indicate longer times to reach the goal box in the TBI group vs. the sham group.

### Histology

All mice were euthanized 15 days after the last mTBI/sham injury by anesthetization with isoflurane, followed by transcardial perfusion with heparinized PBS (pH 7.4) and then by PBS containing 4% paraformaldehyde. After perfusion, brains were post-fixed in 4% paraformaldehyde (4°C) for 48 h. Brains were then blocked paraffin embedded using Tissue-Tek VIP (Sakura, USA), cut at 6 μm on a 2030 Biocut microtome (Reichert/Leica, Germany) and mounted on positively charged glass slides (Fisher, Superfrost Plus). Prior to staining, sections were deparaffinized in xylene, and rehydrated in an ethanol to water gradient. Slides were analyzed under bright field microscope (BX60, Leica, Germany) and digital images visualized and acquired using a MagnaFire SP camera (Olympus, Tokyo, Japan). Sets of adjacent sections were stained for glial fibrillary acid protein (GFAP, 1:20,000; Dako, Glostrup, Denmark, ZO334), ionized calcium binding adaptor molecule 1 (Iba1. 1:5000; Abcam, Cambridge, MA, ab5076), or amyloid precursor protein (APP, 1:40,000; Millipore, Billerica, MA, MAB348). As a negative control, a single section was processed for immunostaining with the exception of the primary antibody. Tissue sections were subjected to antigen retrieval with either heated tris-ethylenediaminetetraacetic acid (EDTA) buffer (pH-8.0) or modified citrate buffer (Dako, Glostrup, Denmark, S1699) under pressure for 7 min. Endogenous peroxidase activity was quenched with a 15 min H_2_O_2_ treatment (3% in water). Tau immunohistochemistry was performed using the following monoclonal antibodies at a 1:500 dilution: AT8 [Ser202, Thr 205] (ThermoFisher Scientific, Waltham, MA MN1020), CP13 [pS202]; PHF1 [pS396/404]; RZ3 [pThr231]. CP13, PHF1, and RZ3 generously provided by Dr. Peter Davies, The Feinstein Institute for Medical Research, Bronx, NY. Each section was rinsed and incubated with the appropriate blocking buffer (ABC Elite kit, MOM kit, Vector Laboratories, CA) for 20 min, before applying the appropriate primary antibody overnight at 4°C. Then, the diluted biotinylated secondary antibody from the ABC Elite Kit was applied. Antibodies were detected using the avidin-peroxidase complex, after incubation with the chromogen 3,3-diaminobenzidine (DAB) peroxidase solution (0.05% DAB−0.015% H_2_O_2_ in 0.01 M PBS, pH 7.2) for 6–7 min and counterstained with hematoxylin. Immunofluorescence was performed with antibodies for the microglial marker Iba1 (1:300), astrocyte marker GFAP (1:5000), and Phospho-syk [Tyr525/526, (1:200) Antibody #2711, Cell Signaling]. Prior to immunostaining, samples were deparaffinization in xylene and rehydration in ethanol solutions of decreasing concentrations (2 × 100%, 95%, 70%). Antigen retrieval was performed by heating slides in a citric acid buffer (Dako, Glostrup, Denmark, S1699) under pressure, washed with PBS and transferred into a Sudan black solution (15 min) to inhibit autofluorescence. Before primary antibody treatment slides were blocked for 1 h with 10% donkey serum. Primary antibodies for Iba1, GFAP, PSyk were applied on the slides and left overnight at 4°C. The next day, secondary antibodies Alexa488, Alexa568 and Alexa647 were applied for PSyk, Iba1, and GFAP, respectively. Slides were cover slipped with ProLong Gold Antifade DAPI Mount.

### Immunohistochemical quantification

Mice from both age groups were euthanized 15 days post-injury. For each animal, sets of sagittal (*n* = 4) sections were stained and analyzed by an observer blinded to experimental conditions using ImageJ software (US National Institutes of Health, Bethesda, MD). Using this software, images were separated into individual color channels (red hematoxylin counter stain and DAB brown chromagen) using the color deconvolution algorithm. Three non-overlapping areas of 100 μm^2^ from each of two sagittal sections in the corpus callosum (CC) were randomly selected within which the area of GFAP or Iba-1 immunoreactivity was calculated and expressed as a percentage of the field of view. The numbers of APP-positive profiles were counted in three non-overlapping areas of 100 μm^2^ within the CC.

The immunohisotochemical outcomes were percent area of GFAP and Iba-1 and the number of APP-positive profiles. The effects of interest in this analysis were sex, injury group (mTBI vs. sham), age (young v aged), and their interactions. Descriptive statistics, including means and standard errors, were calculated from the percent area of GFAP and Iba-1 measurements for each age, injury group, and sex. Average percent areas were calculated within an animal (across sections), prior to calculating age, injury group, and sex averages and standard errors. Descriptive statistics, including medians and 25th and 75th percentiles, were calculated from the number of APP-positive profiles for each age, injury group, and sex. The raw percent area data were assessed for normality using the Shapiro-Wilk test as well as four alternative transformations (square-root, base-10 logarithm, logit, and arcsine square-root). The transformation that closest approached normality was used for all subsequent analysis. Separately for GFAP and Iba-1, the percent area data was fit with the following mixed ANOVA model:

$$\begin{array}{l} Percent\;Area_{ijklm}=\mu +age_{i}+group_{j}+sex_{k}+age_{i}*group_{j}\\ \;+\;age_{i}*sex_{k}+group_{j}*sex_{k}+age_{i}*group_{j}\\ \;*\;sex_{k}+\varepsilon_{ijkl} \end{array}$$

where μ is the overall mean percent area, *age*_*i*_ is the effect of the *i*th age (*i* = Young or Aged), *group*_*j*_ is the effect of the *j*th group (*j* = mTBI or sham), *sex*_*k*_ is the effect of the *k*th sex (*k* = female or male), *age*_*i*_ * *group*_*j*_ is the interaction effect between *age*_*i*_ and *group*_*j*_, *age*_*i*_ * *sex*_*k*_ is the interaction effect between *age*_*i*_ and *sex*_*k*_, *group*_*j*_ * *sex*_*k*_ is the interaction effect between *group*_*j*_ and *sex*_*k*_, and *age*_*i*_ * *group*_*j*_ * *sex*_*k*_ is the interaction effect between *age*_*i*_, *group*_*j*_, and *sex*_*k*_. The ε_*ijkl*_ term represent the leftover difference of the *l*th observation of *age*_*i*_, *group*_*j*_, and *sex*_*k*_ that is unexplained by the model. In addition to these model terms, a random variance component for mouse was included such that multiple observations on the same mouse were weighted together and not individually. This model was used to estimate the size and significance of the difference in percent area between ages, injury groups, and between sexes.

For the APP data, no APP positive profiles were detected in any of the sham animals. As a result, the analysis for these data was subset to the mTBI injury groups. Analysis of the number of APP-positive profiles determined using the following Poisson regression model:

$$\begin{array}{l} Number\;of\;APP-positive\;profiles_{iklm}=\mu +age_{i}+sex_{k}+age_{i}\\ \;*\;sex_{k}+\varepsilon_{ikl} \end{array}$$

where μ is the overall rate of the number of APP-positive profiles, *age*_*i*_ is the effect of the *i*th age (*i* = Young or Aged), *sex*_*k*_ is the effect of the *k*th sex (*k* = female or male), and *age*_*i*_ * *sex*_*k*_ is the interaction effect between *age*_*i*_ and *sex*_*k*_. The ε_*ijkl*_ term represent the leftover difference of the *l*th observation of *age*_*i*_, *group*_*j*_, and *sex*_*k*_ that is unexplained by the model. In addition to these model terms, a random variance component for mouse was included such that multiple observations on the same mouse were weighted together and not individually. This model was used to estimate the size and significance of the difference in the number of APP-positive profiles between ages and between sexes. All statistical analysis of the immunoreactivity data was performed using SAS (ver. 9.4) and all results are reported using the 0.05 level of significance.

## Results

### Neurobehavior

**Figure 1:**
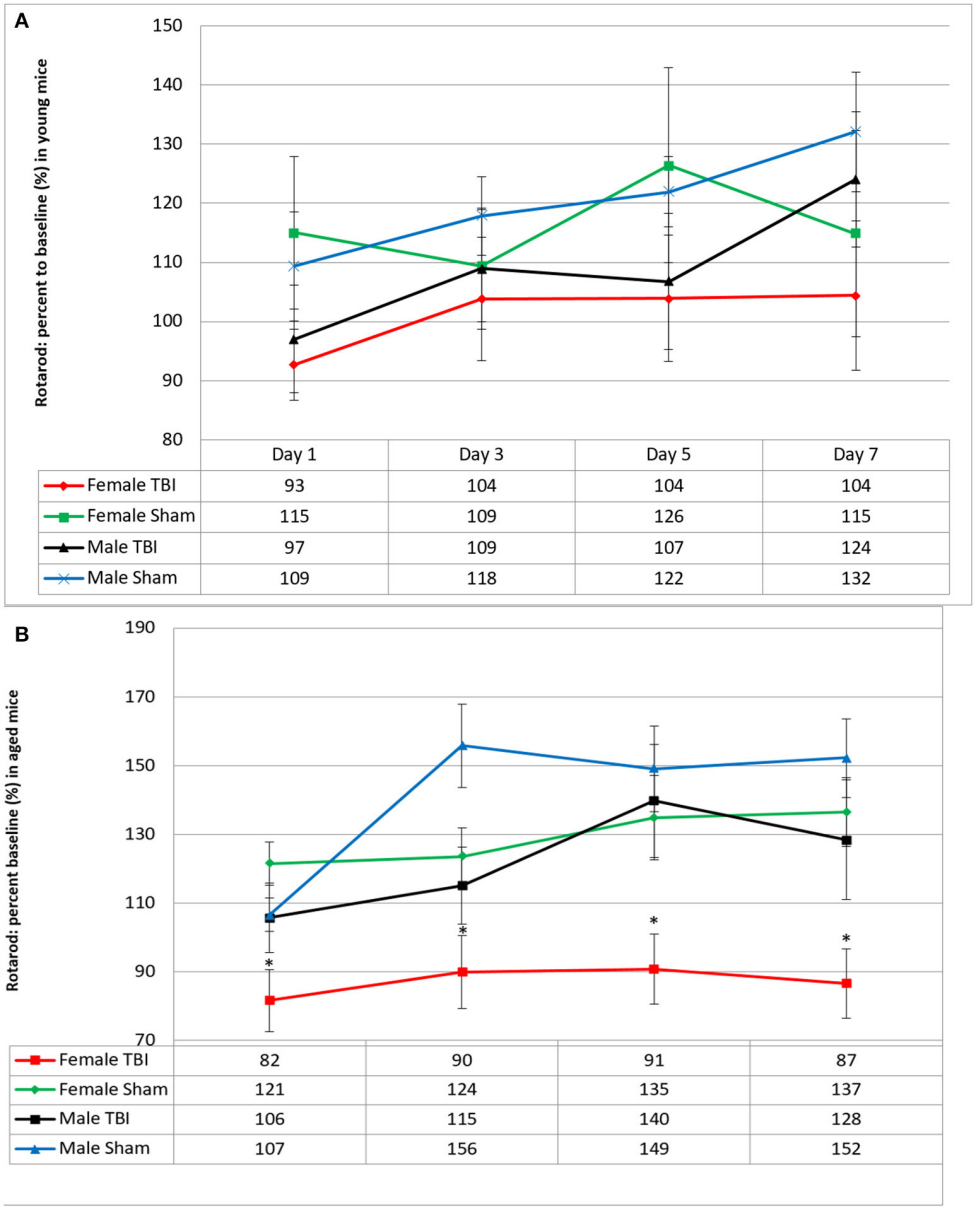
Effect of repetitive mild traumatic brain injury (r-mTBI) on rotarod performance in **(A)** young and **(B)** aged hTau mice. Values were recorded with a total of 4 trials within a week after the last injury/anesthesia with three 5 min accelerating trials. Results are the mean ± SEM of the time animals remained on the rotarod before falling and normalized to baseline. There was no impairment with multiply injured animals in young male and female between days 1 and 7 of testing after last mTBI or anesthesia (*p* > 0.05). In the aged cohort, Female TBI mice performed worse than their sham counterparts (*p* < 0.001) and worse than the male r-mTBI group (*p* < 0.001) on all days of Rotarod testing. ^*^p < 0.05.

Motor function was evaluated 24 h following the last mTBI/anesthesia (Figure 1). Neither young male or female mice had sensorimotor deficits by the end of the trials (male r-sham 132 ± 10%; vs. male r-mTBI 124 ± 11%; and female r-sham 115 ± 17%; vs. female r-mTBI 104 ± 12% on day 7). However, in the aged cohort, female r-mTBI mice showed shorter Rotarod fall times when compared to their female sham counterparts (female r-sham 137 ± 10%; vs. female r-mTBI 87 ± 10%; *p* < 0.001 on day 7) and performed worse than their male r-mTBI counterparts (female r-mTBI 87 ± 10%; vs. male r-mTBI 152 ± 17%; *p* < 0.001 on day 7).

**Figure 2:**
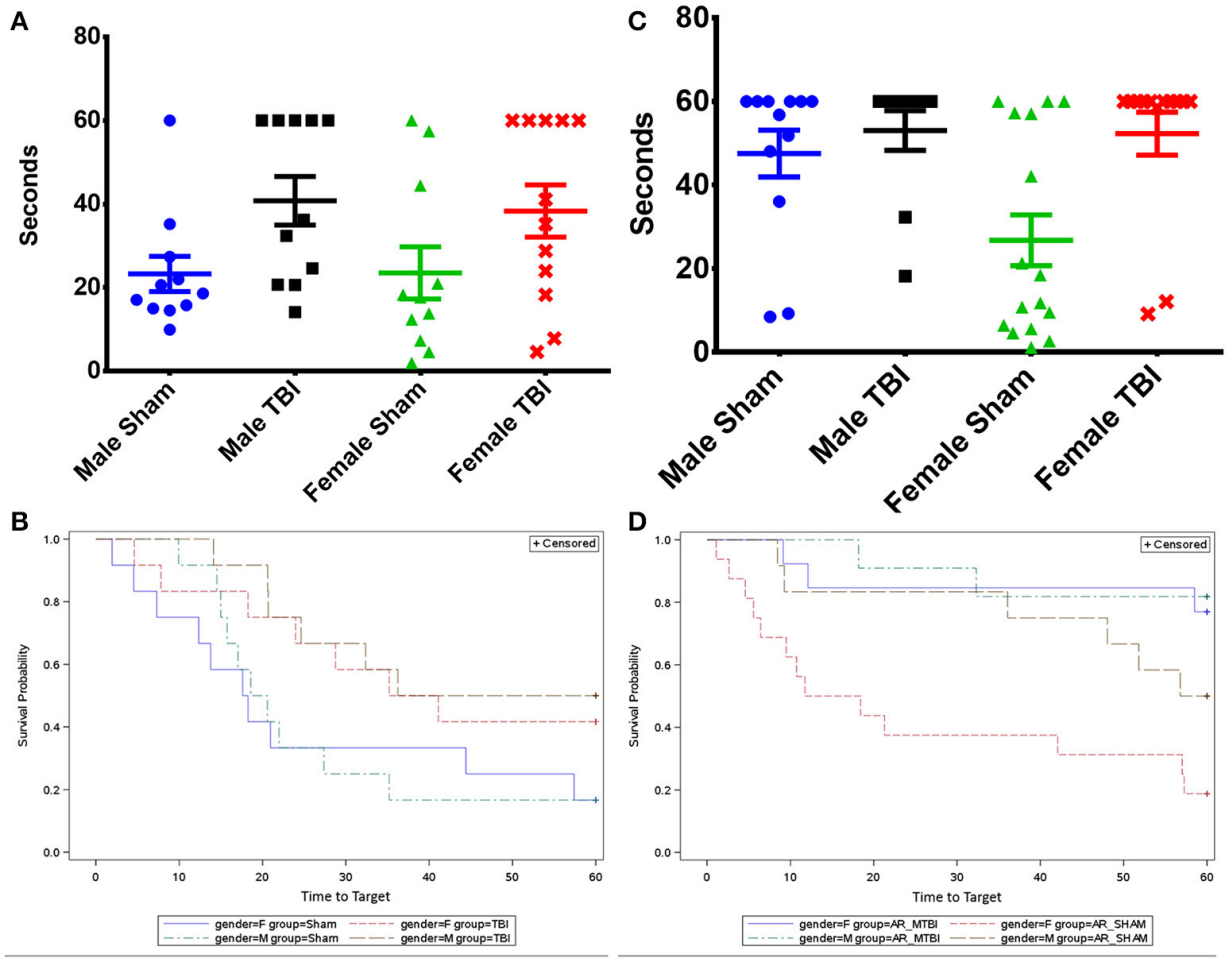
**(A)** Evaluation of spatial memory retention (probe) using the Barnes maze on days 8–14 after last mTBI in young mice. **(B)** Survival analysis chart of the time to find the target hole in the Barnes maze probe trial. **(C)** Evaluation of spatial memory retention (probe) using the Barnes maze on days 8–14 after last mTBI in aged mice. **(D)** Survival analysis chart of the time to find the target hole in the Barnes maze probe trial. Bars represent standard error but are biased by ceiling effects within the data.

Cognitive deficit at 15 days post-last mTBI/anesthesia was evaluated with the Barnes Maze behavioral test. The probe trial analysis of the average time to reach the target zone, (defined by the target escape hole and its adjacent north and south holes) revealed that the performance of the young r-mTBI mice was markedly impaired compared to the sham group when data are combined across sexes (Figure 2, *p* < 0.02). Results of fitting the Cox proportional hazards model indicated no interaction between sexes and injury group (*p* = 0.776), and no sex effect (*p* = 0.726).

For aged mice, the median time to reach the target hole was also lengthened in the r-mTBI group compared to the r-Sham group for females (*p* < 0.004) and for both sexes combined (*p* = 0.001). Results of fitting the Cox proportional hazards model indicated no interaction between sexes and injury group (*p* = 0.499), and only a marginal effect of sex (*p* = 0.063).

### APP immunostaining

**Figure 3:**
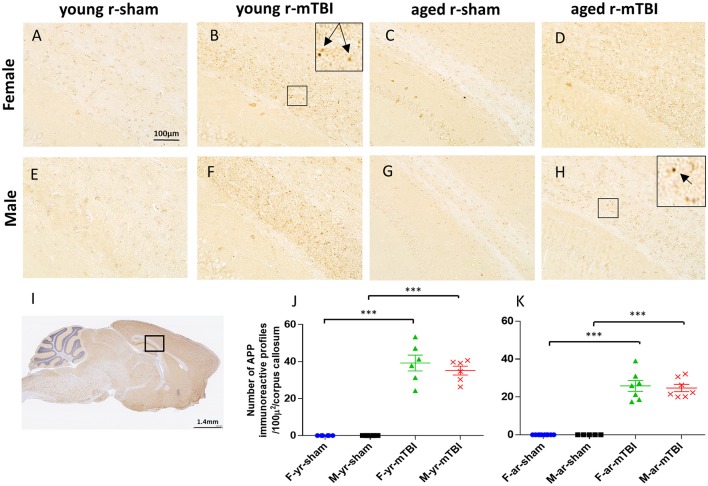
Amyloid Precursor protein (APP) immunohistochemistry of sagittal sections of the mouse brain at ± 0.4 mm lateral to midline in the corpus callosum in female **(A–D)** and male **(E–H)**. Black box **(I)** indicates the area of interest shown at higher magnification **(A–H)** in a sagittal section of a mouse brain. Tissue staining from young and aged sham **(A,C,E,G)** was negative for APP immunostaining. Immunoreactive axonal profiles were observed as discrete axonal profiles in the corpus callosum of all injured animals **(B,D,F,H)**. The number of APP-positive profiles was greater in young vs. aged mice among females (*p* < 0.01) and overall (averaged over sex, *p* < 0.001) **(J,K)**. The interaction between age and sex was not significant. ^***^p < 0.0001.

APP-immunoreactive axonal profiles, a marker of axonal injury, were observed 15 days post-injury in the corpus callosum (CC) (Figure 3I) of both young and aged r-mTBI groups (Figures 3B,D,F,H) but not in their controls (Figures 3A,C,E,G). These APP immunoreactive axonal profiles were observed as small, granular immunoreactive profiles within the CC (Figure 3). There were no APP-positive profiles observed in the Sham animals. Analysis found that the number of APP-positive profiles was significantly greater in young vs. aged mice among females (*p* = 0.0016) and overall (averaged over gender, *p* = 0.0005), but the effect was only marginally significant among males (*p* = 0.0643) (Figures 3J,K). The interaction between age and gender was not significant, which indicates that age effects can be combined across genders and gender effects can be combined across ages. There were no significant gender effects detected.

### GFAP immunostaining

**Figure 4:**
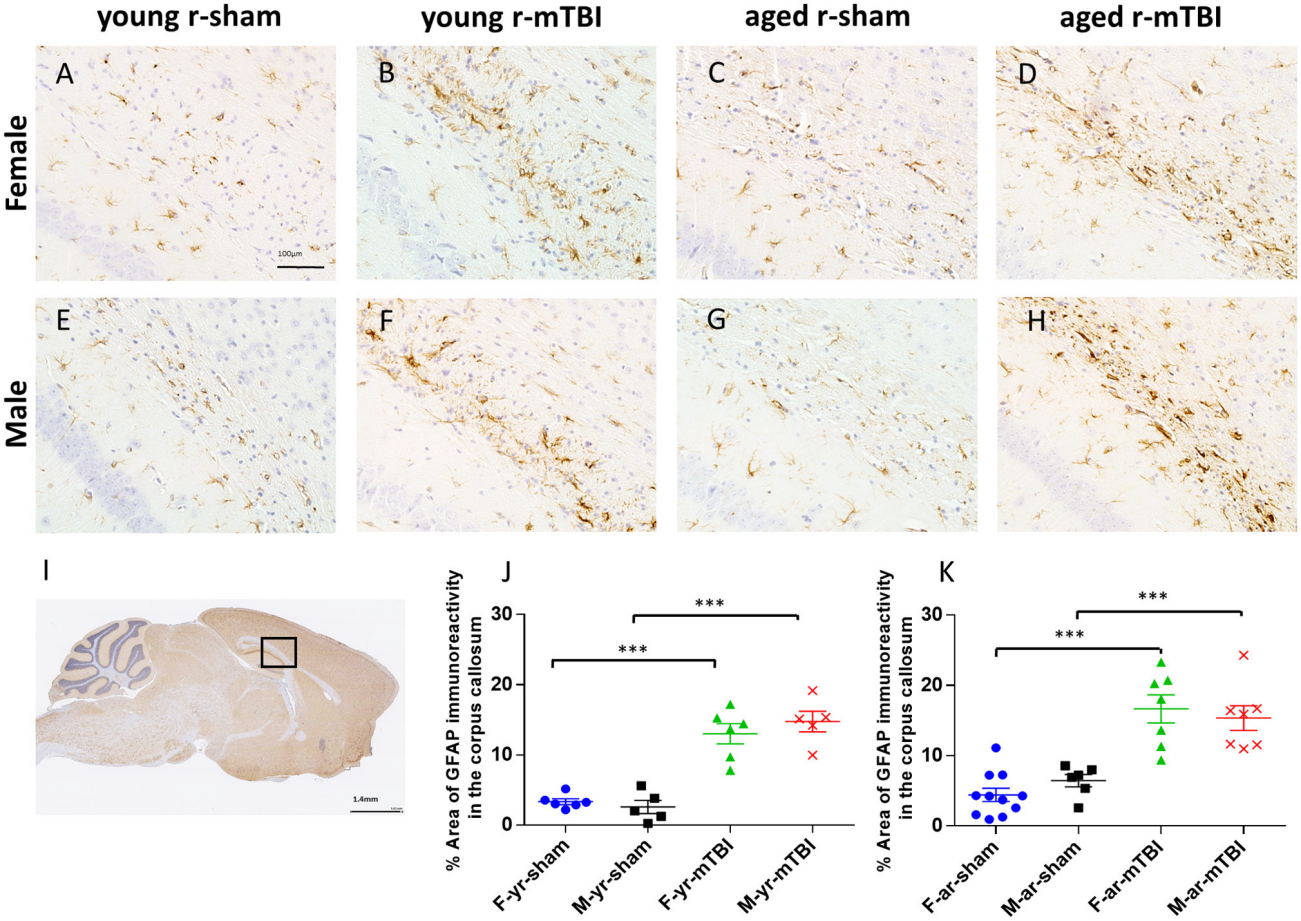
Glial fibrillary acid protein (GFAP) immunohistochemistry of sagittal sections of the mouse brain at ± 0.4 mm lateral to midline in the corpus callosum in male **(A–D)** and female **(E–H)**. Black box **(I)** indicates the area of interest shown at higher magnification **(A–H)** in a sagittal section of a mouse brain. Healthy astrocytes with quiescent morphology were observed in sham animals in both young and aged mice. The average percent area for the r-mTBI group was greater compared to the sham control group for females (averaged over age, *p* < 0.0001), males (averaged over age, *p* < 0.0001), aged (averaged over sex, *p* < 0.0001), young (averaged over sex, *p* < 0.0001), and overall (averaged over sex and age, *p* ≤ 0.0001) **(J,K)**. Additionally, the average percent area for the young mice was lower compared to the aged mice for females (averaged over injury group, *p* = 0.0423) and overall (averaged over sex and injury group, *p* = 0.0077). Tissue sections were counterstained with hematoxylin. ^***^p < 0.0001.

The effect of mTBI and sex on astroglial activation was evaluated at 15 days post-injury in both young and aged animals in the body of the CC (Figure 4I). For mice subjected to r-mTBI, immunostaining for GFAP revealed evidence of a reactive astrogliosis in the CC of both aged groups (Figures 4B,D,F,H; *p* < 0.0001). Astrocytes with quiescent morphology were observed in sham animals at both time points (Figures 4A,C,E,G). No sex effect was observed among the sham or injured groups at 15 days post-injury (*p* > 0.05). Additionally, the average percentage area of astroglial activation for the young mice was lower compared to the aged mice for females (averaged over injury group, *p* < 0.05) and overall (averaged over sex and injury group, *p* < 0.05) (Figures 4J,K).

### Iba1 immunostaining

**Figure 5:**
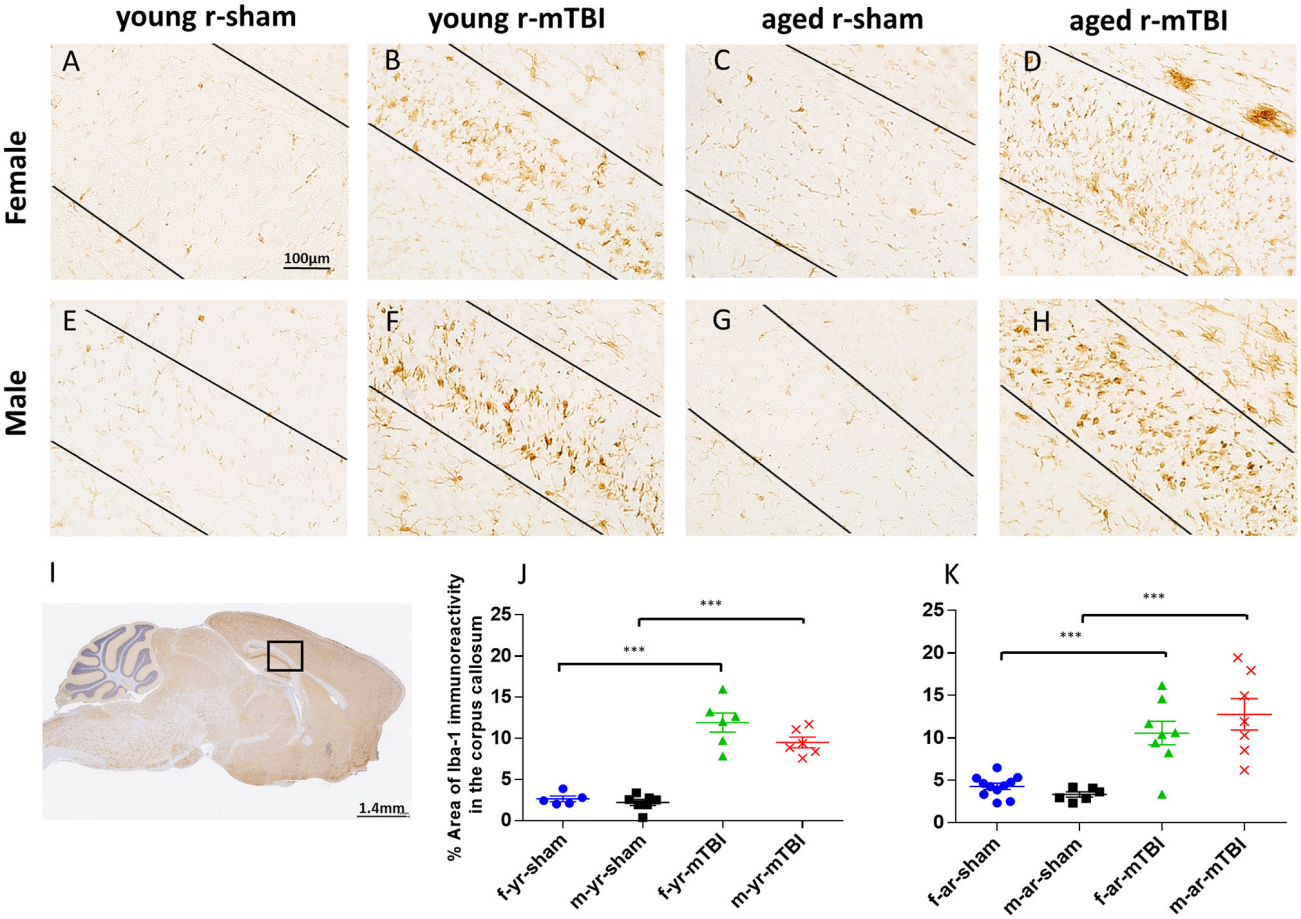
Immunohistochemical labeling for microglia with anti-Iba1. Sagittal sections of the mouse brain at ± 0.4 mm lateral to midline in the corpus callosum in female **(A–D)** and male **(E–H)**. Black box **(I)** indicates the area of interest shown at higher magnification **(A–H)** in a sagittal section of a mouse brain. Black lines indicate the approximate boundary of the corpus callosum body within each image. There was no microglial activation in the sham groups **(A,C,E,G)**. An increased area of anti-Iba1 immunoreactivity was observed in the corpus callosum at 15 days post r-mTBI in young and aged animals **(B,D,F,H)**. The average percent area for the young mice was lower compared to the aged mice (averaged over injury and sex, *p* < 0.05, **J,K**). Additionally, the average percent area for the young mice was lower compared to the aged mice for males (averaged over injury group, *p* < 0.05; **J,K**). ^***^p < 0.0001.

The pattern and distribution of activated microglia revealed in staining for Iba-1 was similar to GFAP. In the sham animals, none of the regions of interest showed cells with structural characteristics of activated microglia (hypertrophic and bushy morphology); (Figures 5A,E,C,G; *p* > 0.05). However, in the CC (Figure 5I), the r-mTBI mice showed a notable increase in Iba1-immunoreactivity with clusters of activated microglias (Figures 5B,F,D,H; *p* < 0.0001). Additionally, the average percent area of Iba1-immunoreactivity for the young mice was lower compared to the aged mice for males (averaged over injury group, *p* < 0.05), young sham mice were significantly lower than the aged sham group (averaged over sex, *p* < 0.05), and overall (averaged over sex and injury group, *p* < 0.05) (Figures 5J,K). There were no sex effects detected (*p* > 0.05). The quantification of GFAP and Iba1 immunostaining is summarized in Table 1.

**Table 1:** Summary of GFAP and Iba1 quantification.

	**Age of Mice**	**Gender**	**Treatment Group**	**Mean Percent Area**
				**(Std. Error)**
GFAP	Young	Sham (Control)	Female	3.35 (0.41)
			Male	3.05 (0.90)
		mTBI	Female	11.18 (2.21)
			Male	14.32 (1.26)
	Aged	Sham (Control)	Female	4.50 (0.92)
			Male	6.31 (0.94)
		mTBI	Female	16.13 (1.80)
			Male	16.09 (1.90)
Iba1	Young	Sham (Control)	Female	2.65 (0.34)
			Male	2.21 (0.36)
		mTBI	Female	11.92 (1.16)
			Male	9.51 (0.65)
	Aged	Sham (Control)	Female	4.28 (0.37)
			Male	3.34 (0.31)
		mTBI	Female	10.56 (1.39)
			Male	12.76 (1.85)

### Tau immunohistochemistry

**Figure 6:**
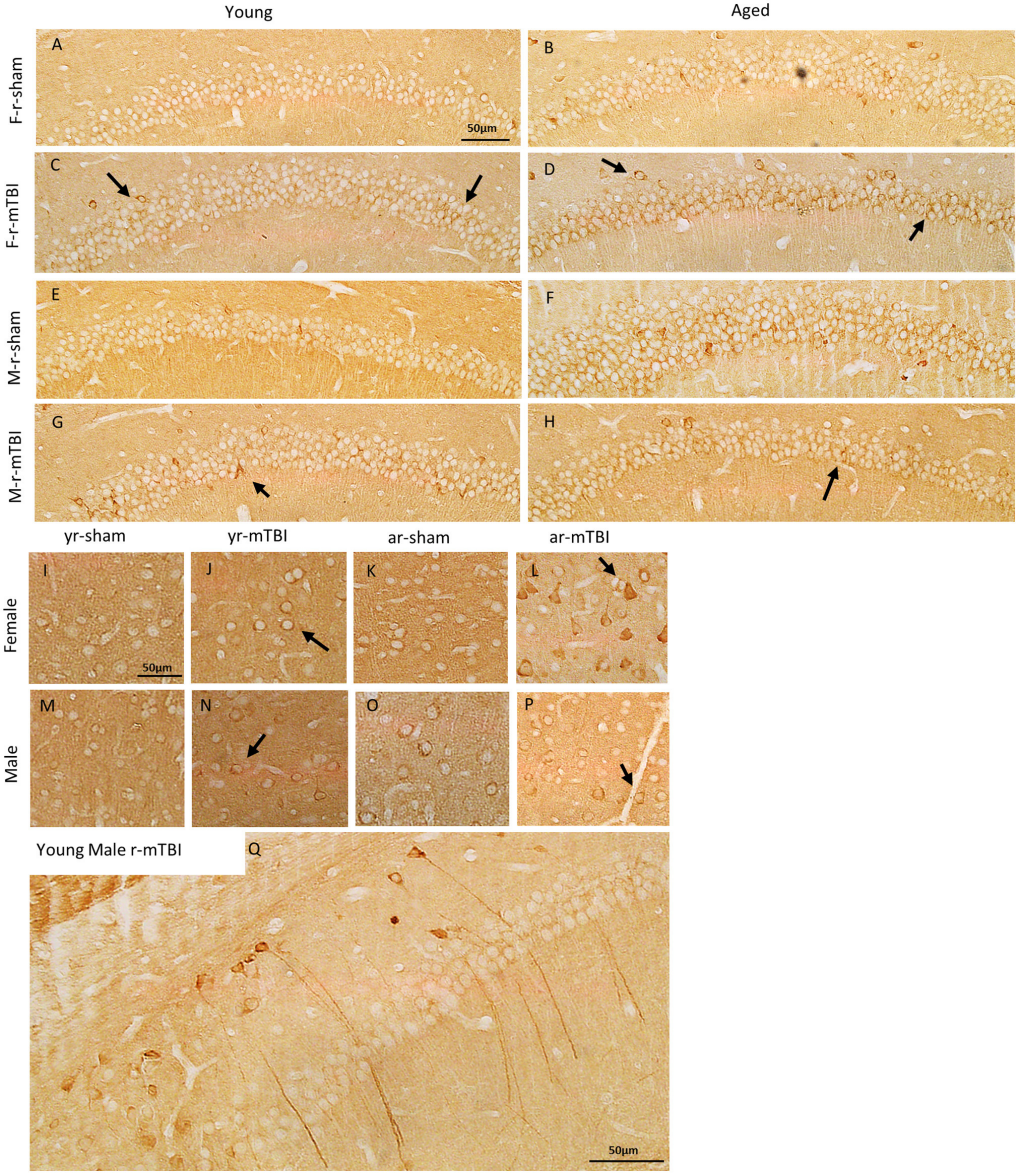
Immunohistochemical assessment of soluble phosphorylated tau pSer-202 (CP13) at approximately 0.5 mm lateral to midline in the CA1 region of the hippocampus **(A–H)** and in the neocortex **(I–P)** at 15 days after injury in young and aged female and male mice. Qualitatively, the r-mTBI group showed greater dendritic and membranous staining in both the CA1 (arrows in **C,D,G,H**) and cortical neurons (arrows in **J,L,N,P**) when compared to their respective shams. In in the aged group, intraneuronal immunoreactivity for CP13 was generally more intense within the perikarya and proximal apical dendrite of neurons in the hippocampal CA1 field of the male r-sham group **(F)**. In addition, regardless of their sex and aged, some of the injured mice showed more intense CP13 immunoreactivity in long dendritic process when compared to their respective sham **(Q)**.

To investigate the effect of TBI on tau in this model, we performed immunohistochemical analyses using CP13, RZ3, AT8, and PHF1 (an antibody that recognizes early tau pathology). Immunohistochemical analyses revealed no sex or aged effect in the localization or accumulation for the p-tau epitopes CP13 and RZ3. However, all injured groups exhibited somatodendritic accumulation of CP13 immunoreactivity in the hippocampal subfields CA1 (see Figures 6A–H), and CA3 (data not shown) as well as in the neurons in the superficial layer of the cerebral cortex (see Figures 6I–P). Additionally, intense dendritic staining was noticed in some injured mice regardless of sex or age (see Figure 6Q). Interestingly, intraneuronal CP13 immunoreactivity in aged male sham mice was generally more intense than the aged females but only in the CA1 subregion of the hippocampus. The same brain region was devoid of neurons positive for RZ3, PHF1, and AT8 regardless of the injury status (see Supplemental Figure 1).

**Figure 7:**
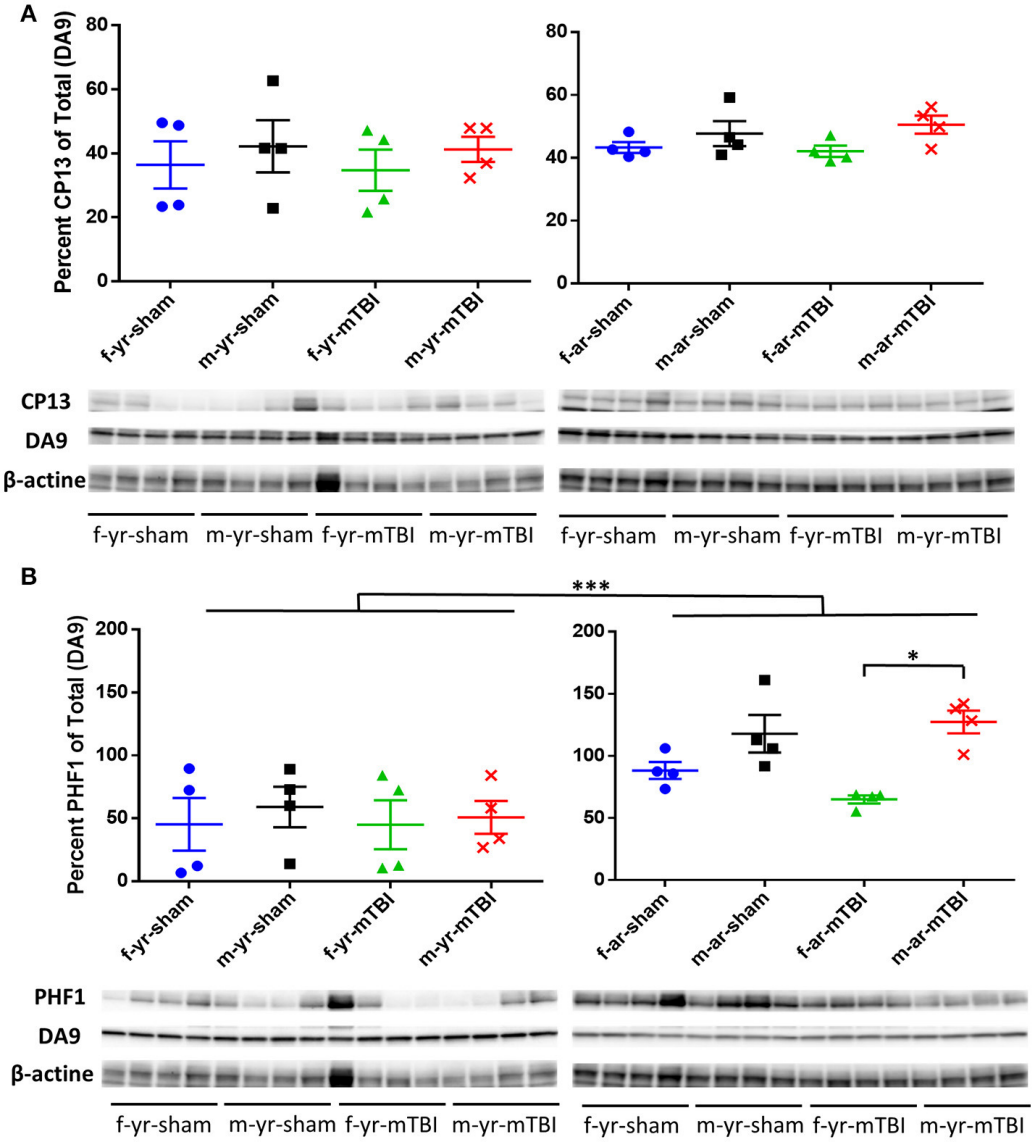
Immunoblots of cortical brain extracts of mice probed with monoclonal antibody CP-13 (pS202/pT205), PHF1, (pS396/404) or β-actine at 15 days post-injury in young (left panels) or aged animals (right panels). For the CP13 data **(A)**, there were no difference observed between gender, age, or treatment (^*^, *p* > 0.05). For the PHF1 data **(B)**, the average percent of PHF1 for the young mice was lower compared to aged mice averaged over gender and treatment (*p* < 0.0001) as well as within female mice (averaged over treatment, *p* = 0.0362), within male mice (averaged over treatment, *p* < 0.0001), within the mTBI treatment group (averaged over gender, *p* = 0.0024), and within the sham treatment group (averaged over gender, *p* = 0.0015). Additionally, the average percent of PHF1 among female mice was lower compared to the male mice averaged within the mTBI treatment (averaged over age, *p* = 0.0244). All densitometry values for CP13, PHF1, DA9 for individual bands were normalized to the beta actin (β-actin) value for their respective lane and this ratio used for statistical analysis. The primary outcomes in the Western Blot analysis were the percent of CP13 and PHF1 antibodies relative to the total (DA9). *N* = 4 (injured/sham). ^*^p < 0.05; ^***^p < 0.0001.

For the CP13 western blot data, there were no significant differences observed in the percent of total data between treatment, gender, or age nor were any interactions between age, treatment group, or gender detected. For the PHF1 western blot data, significantly lower percent of total values were observed in the young mice, compared to aged mice, within both treatments, within both age groups, and averaged over treatment and age (Figure 7). Additionally, significantly lower percent of total values were observed in female mice, compared to male mice, within the mTBI treatment group, within aged mice, and averaged over age and treatment. No interactions between age, treatment group, or gender were detected in the PHF1 data.

### Phospho Syk immunohistochemistry

**Figure 8:**
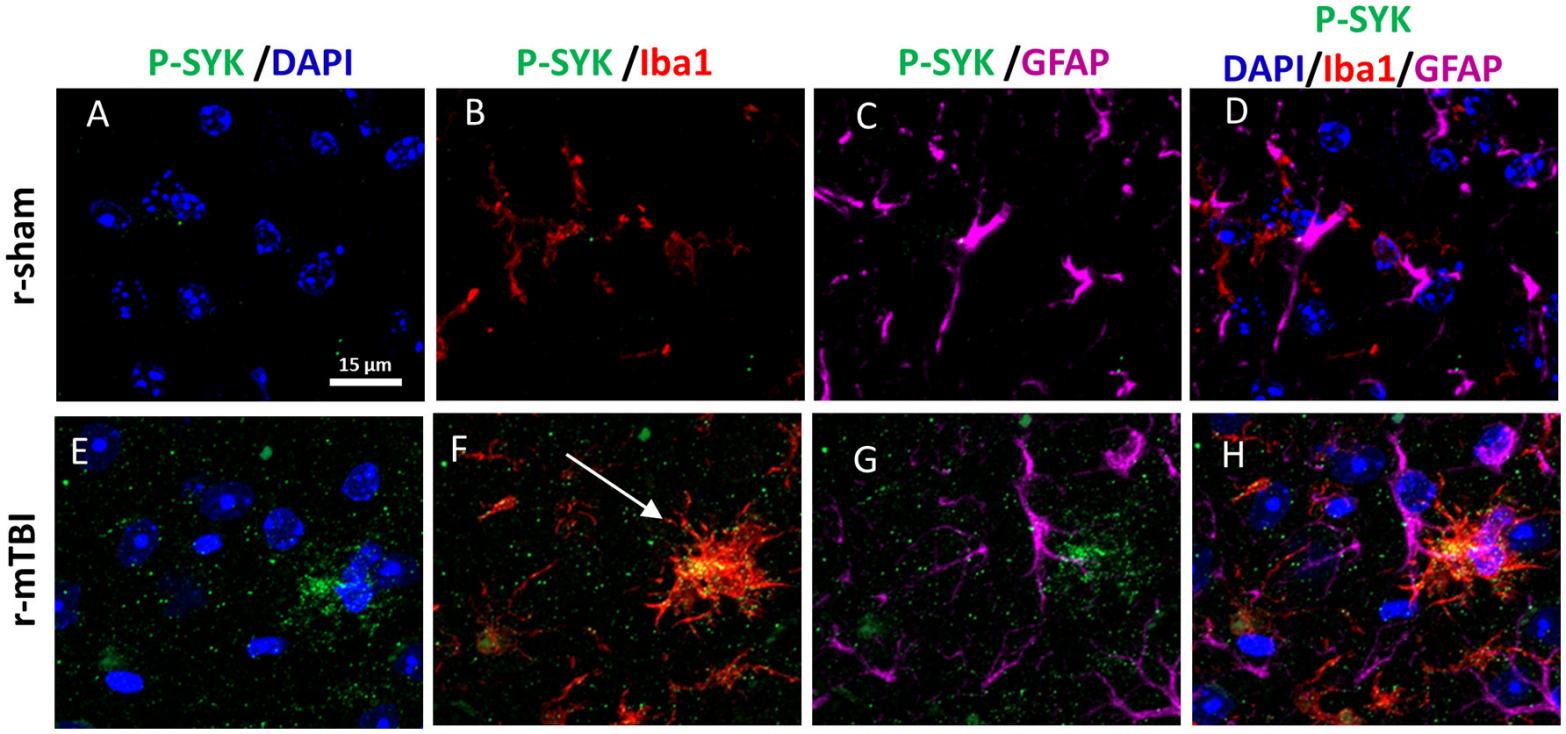
Immunohistochemical labeling for P-SYK, anti-Iba1 and GFAP. Sagittal sections of the mouse brain at ± 0.5 mm lateral to midline in the corpus callosum in aged male animal **(A–H)**. Sham mice show an absence of P-SYK immunoreactivity (green fluorescent signal) in the corpus callosum **(A–D)** whereas r-mTBI mice demonstrate the presence of activated SYK **(E–H)** in microglia (white arrow, **F**). Of note, GFAP and Iba-1 immunoreactivity is increased in r-mTBI compared to sham mice revealing astrogliosis and microgliosis.

Our previous work on Alzheimer Disease (AD) demonstrated that Syk regulates Tau phosphorylation by controlling the activation of glycogen synthase 3β (Paris et al., 2014), thus sections were immunostained for p-Syk. At 15 days post-injury we observed in both young and aged injured animals an increase of p-Syk immunoreactivity in activated microglia within the corpus callosum regardless of their sex (Figure 8), while p-Syk immunoreactivity was absent in their respective shams.

## Discussion

In the current study, we have examined the effect of sex on neurobehavioral and pathological outcomes in young and aged hTau mouse after repetitive mild TBI. Our results demonstrate that young (aged 8–10weeks) animals exposed to r-mTBI have short-term behavioral abnormalities that manifest as impaired spatial memory, but no deficits in motor function. The injury used in the experiment is mild in nature, producing no skull fracture, bleeding, or other gross pathology and requires minimal time under anesthesia since no incision is necessary. No sex-specific effects were observed during neurobehavioral testing of young animals. In contrast, in aged animals we observed impaired motor coordination in the Rotarod task and spatial memory in the Barnes maze task exclusively in female TBI mice, in keeping with an age-dependent effect of sex on outcomes from r-mTBI in this model.

In the Rotarod test, aged female mice exclusively exhibited impaired motor performance after TBI. This effect does not appear to correlate with either the neuroinflammatory nor the tau pathology seen in these animals, but may be driven by effects in the vestibular nuclei that we have yet to be investigated. Future studies will examine additional pathological markers across additional brain regions and at additional time points post-injury, to better understand these sex-specific differences in the response to TBI in the aged brain. Relevant TBI-dependent pathology may reside, for instance, in regions of the basal ganglia, not yet analyzed, and may require analysis of markers not yet employed by our studies. For example, Uryu and colleagues have previously reported transient increases in synuclein pathology that were specific to aged mice, with increases in alpha and beta synuclein present 1 week following TBI with antibodies for conformationally altered and nitrated both showing positive staining within the striatum (Uryu et al., 2003). Peripheral nervous system deficits may also be a contributing factor in these results; TBI is known to be associated with motor neuron disease-like peripheral pathology (Wright et al., 2016) and mild closed head injury has been shown to exacerbate rotarod deficits and increase markers of denervation in a G93A transgenic mouse model of ALS (Evans et al., 2015). Although sex-differences have not been shown as a factor in these studies, our findings suggest that it may be an important area for future studies to examine.

Neurobehavioral testing of spatial memory did not show any sex-dependent effects of TBI in young mice, where TBI impaired the spatial memory of male and female mice equally at 15 days post-injury, but in the aged mice a TBI-dependent effect on Barnes maze testing was specific to female mice. The absence of a TBI effect in the male mice was driven by worsened performance of male sham mice. To our knowledge, this age and sex-dependent effect on Barnes maze spatial memory has not been previously reported in hTau mice (P. Davies, pers. comm.). Aged male sham, male TBI, and female TBI mice all exhibited qualitatively higher levels of perikaryal tau immunoreactivity by CP13 in the hippocampus than female sham mice, potentially explaining the differences in spatial memory that we observed in these aged mice.

Young hTau mice exhibited a pronounced r-mTBI dependent astroglial/microglial activation in the corpus callosum of both male and female htau mice, indicating no sexual dimorphism for the glial response at 15 days post-injury. The effect of sex and age on white matter degeneration was similar among all groups suggesting that male and females may be equally susceptible to axonal injury. While multiple reports have noted that female rodents appear to be protected against the acute CNS trauma and stroke (Roof and Hall, 2000b; Dang et al., 2011; Wright et al., 2011), we report no effect of sex on the glial response or axonal transport interruption in our study. In contrast, our data are in agreement with the recent study of Villapol et al, where female mice are acutely more resistant to moderate to severe TBI compared with male mice during the acute and subacute phase (3–7 days) post-injury but comparable to their male counterpart at 30 days post-injury (Villapol et al., 2017). Future studies will be conducted to determine if there are any sex-dependent histological differences in these hTau mice at shorter post-injury timepoints. We further report that, while no sexual dimorphism was observed in tau pathology in the young animal, a trend for an increased phopho tau pathology in neuronal cell body and somatodendritic compartments of the aged sham male comparable to the injured animals. Western blots showed no sexual dimorphism in the cortical levels of CP13 in young or aged mice, but did reveal significantly lower PHF1 levels in aged female TBI mice compared to male TBI mice, validating sexual dimorphisms of phospho tau levels in these mice. While more studies are required to establish whether an increase of CP13 in the underlying hippocampal region and PHF1 in the cortex can be harnessed to worse cognitive outcome, this is the only observation that corroborates with the impaired performance of the aged male sham group. This observation is supported by data from Rubenstein et al also using a model of closed head injury in mice, suggesting that there is a link between cognitive dysfunction and an increase of p-tau (Rubenstein et al., 2017). The tyrosine kinase Syk is known to phosphorylate tau (Lebouvier et al., 2008) and in this study we have found phosphorylated Syk exclusively in TBI mice colocalized with Iba1. In this study, microglial Iba1 pathology associated with injury and age, but no sexual dimorphisms were observed and are unlikely to be the source of the differential phospho tau levels seen in our male and female mice.

While the reduced tau pathology and improved spatial memory of aged female sham mice may suggest a neuroprotective role of female sex, this effect appears to be limited to the age-dependent tau pathology and does not protect against TBI-dependent alterations. Additionally, TBI-dependent motor deficits observed in Rotarod testing of aged female hTau mice demonstrates that the neuroinflammatory and phospho-tau markers examined in the cortex, corpus callosum, and hippocampus as part of this study do not provide a complete picture of TBI-dependent deficits which may be mediated by additional cortical or vestibular changes we have yet to examine. In humans, both peripheral and central damage to the vestibular system is known to occur at various injury levels and produces some of the most common side effects of mild TBI, including dizziness and disruptions to coordinated eye movement (Gizzi et al., 1996; Balaban et al., 2016; Wallace and Lifshitz, 2016; Hoffer et al., 2017). Although most pathology seen in our model of r-mTBI was localized to the corpus callosum, we saw no effect of sex on any of the markers under analyses. Damage to the vestibular system, whether central or peripheral, is far less studied in the pre-clinical literature, though it has been previously discussed as a source of TBI-dependent differences in rotarod performance, and transient increases in glucose metabolism has been observed in the vestibular nuclei after blast TBI in rats (Awwad et al., 2015; Guley et al., 2016).

In this study, we have found age-dependent sex-specific differences in the response to repetitive mild TBI in hTau transgenic mice. Young mice exhibited no sex-dependent differences in behavioral or pathological outcomes at 15 days post-injury. However, aged female mice revealed worse motor outcome after injury compared to males, independent of neuroinflammatory or tau pathology. Conversely, the Barnes maze impairments and increased tau phosphorylation found in aged male mice shows a potential correlation between spatial memory and age-dependent phospho tau pathology. These findings underscore the need for further study of how sex-dependent effects of TBI change with age, and it is important to note that the tau-centric nature of the sexual dimorphisms of our findings suggest that these results may be specific to the hTau mice used. There is a pressing need for additional studies both pre-clinically and clinically to understand how these factors influence TBI outcomes, and to improve our understanding of sex-dependent TBI outcomes in additional mouse strains and genotypes, including wild type animals which may not exhibit these effects. Preclinical models need to adequately study the variables influencing TBI-pathogenesis in order to effectively serve as platforms for discovery and development of therapeutics, and our data suggest that sex-dependent effects on cognition with aging and TBI may be driven in part by differences in sex-dependent tau phosphorylation.

## Author contributions

FC conceived and designed the research. CiL, CaL, AM, SF, and BM performed experiments on mice. SF, BM, GC, BC, and GB analyzed data and prepared figures. SF and BM prepared the initial draft; WS, FC, EM, and MM revised the manuscript. All authors reviewed the final manuscript and approved its publication.

### Conflict of interest statement

The authors declare that the research was conducted in the absence of any commercial or financial relationships that could be construed as a potential conflict of interest.
