# Supplementary material for: Negative Impact of Female Sex on Outcomes from Repetitive Mild Traumatic Brain Injury in hTau Mice Is Age Dependent: A Chronic Effects of Neurotrauma Consortium Study
Source: Front Aging Neurosci. 2017 Dec 22;9:416. doi: 10.3389/fnagi.2017.00416 (PMC5744460; doi:10.3389/fnagi.2017.00416)
Supplement: Supplementary file 1 [file Image1.pdf]

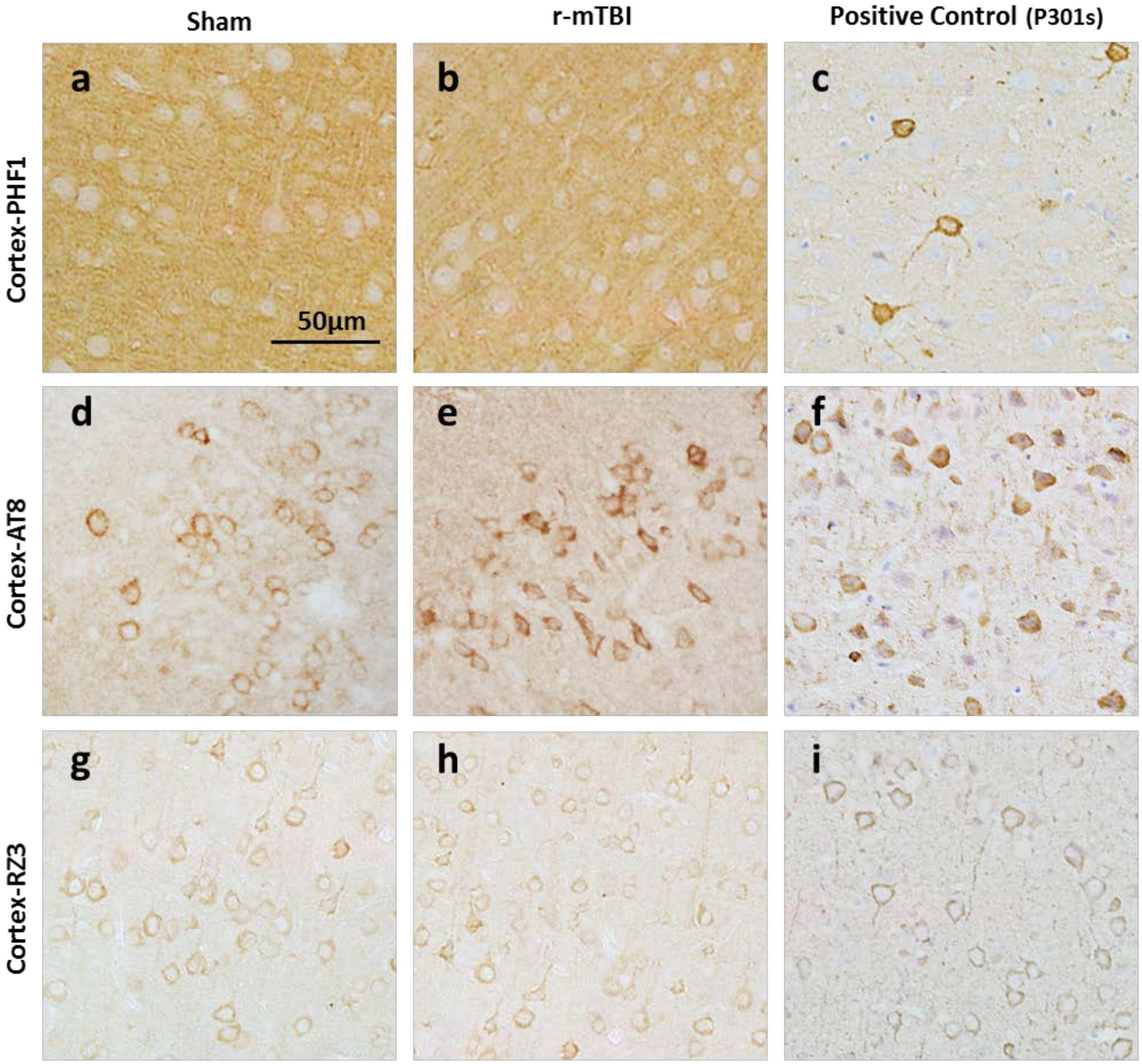

Supplemental Figure 1. Immunohistochemical assessment of phosho-tau antibodies and neurofibrillary tangles in aged hTau male animals at 15 days post injury. We observed no age or gender effect in pTau Ser 396/404 (PHF1) (a,b), pTau Ser 202, Thr205 (AT8) (d,e) or pTau pT231 (RZ3) (g,h) immunostaining in the cortex (and in the CA1 or CA3 sub-region of the hippocampus) of injured compared to sham animals. Immunostaining for PHF1, AT8 and RZ3 from a P301L mouse is shown in the right panels (c, f, i) for positive comparison.
